# Development of an Arabic test for assessment of semantics for the Arabic-speaking children: the Arabic semantic test

**DOI:** 10.1186/s43163-023-00405-3

**Published:** 2023-03-14

**Authors:** Dina Mohammed Abd-Elmoneim, Hassan Hosney Ghandour, Dina Ahmed Elrefaie, Mona Sameeh Khodeir

**Affiliations:** Unit of Phoniatrics, Otorhinolaryngology Department, Faculty of Medicine, Ain Shams University, Abbassia, Lotfy Elsayed Street, Cairo, 11566 Egypt

**Keywords:** Semantics development, Lexical semantics, Arabic-speaking Children

## Abstract

**Background:**

Semantics is the study of meaning, and it pertains to the meanings of linguistic expressions such as words, phrases, grammatical forms, and sentences. Studying semantic development for preschool children has several applications in research design, assessment, and intervention. In English and most Indo-European languages, there is a long tradition of examining aspects of child language by computing different developmental indices from spontaneous language samples and by applying different language tests. However, for the Arabic language, these aspects are lacking in this valuable area of research. Thus, this study aimed to develop and standardize a comprehensive test for assessing semantic language development suitable for preschool children in Arabic-speaking countries. The constructed Arabic semantic test (AST) was administered to 120 typically developed Egyptian Arabic-speaking children between the ages of 2 to ≤ 4 years divided into 4 age groups, with a 6-month age interval between each group. Children’s responses were statistically analyzed to assess the test’s validity and reliability. Ranks of percentiles were calculated to describe the semantic language development in preschool Egyptian Arabic-speaking children.

**Results:**

A statistically significant difference was found over the scores of the Arabic semantic test in both receptive and expressive aspects of semantics among the 4 participating age groups. Semantic growth was positively correlated to the chronological age of the participating children, with good validity and reliability of the test.

**Conclusions:**

The Arabic semantic test is a valid and reliable test that can be applied to assess semantic development among preschool Arabic-speaking children.

## Background

Language content or semantics is the study of meaning [1], and it is concerned with the meanings of linguistic expressions such as words, phrases, grammatical forms, and sentences [2]. Semantics is divided into 3 subfields: lexical, grammatical, and conceptual semantics [3]. Lexical semantics focuses on “content” words rather than “grammatical” words. Thus, it is the meaning of words that belong to one of the four lexical word classes: nouns, verbs, adjectives, and adverbs, and the relation between these classes (lexical relations), while grammatical semantics studies aspects of meaning which have direct relevance to syntax. It includes the meaning of the function words and inflectional affixes, grammatical functions, and the meaning of different sentence types. Conceptual semantics studies the relationship between natural language and formal logical systems, and its fundamental goal is to describe how humans express their understanding of the world using linguistic utterances [4].

During the stage of language development, vocabulary learning is built from a child’s knowledge about objects, actions, locations, properties, and stages gained because of sensory-motor development. Most children usually become competent speakers of their native language by the time they are 5 years of age. Accordingly, during these early years of development, children acquire the ability to (1) perceive and produce the speech sounds of the language to which they are exposed and the phonological rules for combining them to create words, (2) acquire a large and varied vocabulary, and (3) acquire the rules for combining vocabulary into complex grammatical sentences with correct morphology [5]. There are three basic factors underlying children’s word learning according to Bloom [6]: (a) an understanding of mental states (i.e., social–cognitive understandings), (b) an understanding of the kinds of things that get labeled (i.e., cognitive biases), and (c) understanding syntactic clues to word meaning (i.e., syntax).

Word learning begins months before children speak their first words, and comprehension vocabulary is acquired earlier and grows faster than production vocabulary. At 16 months of age, children have a comprehension vocabulary of about 92 and 321 words. However, production vocabularies are typically 0 at 10 months and under 50 words at 16 months [7]. Children usually produce their first words sometime between 10 and 15 months of age. Children’s first words may always be situation-specific or function-specific understandings of word use which is unlike adults’ mental representations of words as symbols that refer to [7]. Children use either way to start their first production vocabularies; this is determined by several factors:

1. Contexts in which children hear the words (words taught as labels and given explicit definitions may be more likely to be used referentially from the beginning than words the child picks up from context)
2. Children’s different approaches to the language acquisition task may reflect the number of context-bound words in early children’s lexicons.
3. The extent to which children are risk-takers; risk-takers may jump into talking with a minimal understanding of what they are saying, whereas cautious children do not use words until they are sure of what they mean so that they are expected to produce fewer context-bound words
4. Sociability: A sociable child will want a vocabulary of words to use in particular situations, is more driven to use whatever means are available for interaction, and he/she is more likely to talk when his or her understanding of word meaning was still incomplete and tied to particular settings. However, a less sociable child has little use for words that serve only a social function and so may wait longer to talk, at which point the child’s understanding of word meaning is more likely to allow contextually flexible usage [7].

As soon as the first words appear, children start adding words to their vocabulary. The number of acquired vocabulary according to age increases from slow growth to rapid growth as the child reaches the age of 2 years. Children can express 20 vocabularies by the age of 18 months; this number rapidly jumps to 200 words at the age of 21 months and reaches 300–400 words as they reach 24–36 months [8]. Researchers identified individual differences in vocabulary development as some children reach the 50- and 100-word milestones at younger ages than others, and the vocabularies of children of the same age vary enormously in size. This difference could be attributed to environmental factors and child factors [7]. The environmental factors involve the amount, and the nature of speech children hear, the child’s birth order and socioeconomic status, the informativeness of the context in which mothers present new words and describe the meaning of every newly added word, maternal verbal responsiveness, or mother-child mutual engagement support word learning, particularly in children under 18 months. The child’s factors include the child’s joint attention skills, personality (very outgoing children may elicit more input), and temperament that results in the child being attentive to his/her environment and easy for others to interact with may promote language development [9] and the child’s cognitive and language skills [10]. Moreover, many studies have demonstrated a relation between phonological memory capacity and concurrent vocabulary size in children from 22 months up to 9 years [11]. Being able to remember how newly encountered words sound a prerequisite would be to add those words to one’s mental lexicon [7].

A striking shift has taken place over the last decade in topics that concern investigators of child language. Studies in the early times concentrated primarily on the acquisition of formal syntactic configurations and operations (e.g., phrase structures, transformations). More recent analysis, in contrast, reflects a growing concern for the way form is related to meaning in linguistic development and more generally for cognitive bases of language acquisition. A test of vocabulary knowledge could require that the child should be familiar with the language’s words to answer each item correctly. The child must know the meaning of the words according to his/her age to be successful [12].

Assessing the vocabulary knowledge of a child can be done on one of two bases: expressive vocabulary or receptive vocabulary. Receptive language plays an important role in the development of expressive language, but expressive language does not influence the development of receptive language to the same degree. Despite these differences, receptive and expressive language skills are highly correlated, such that a relatively high score on a test of receptive language predicts a relatively high score on a test of expressive language [13]. Testing expressive vocabulary might be done by asking the child to provide a name for pictures, while testing receptive vocabulary can be done by asking the child to match spoken words with pictures or a child might be asked to tell a synonym or an antonym for words; this is considered a test of both receptive and expressive vocabulary.

Also, the assessment of semantics could be done at two levels: word and sentence levels. Word level includes identification of synonyms (same meaning), antonym (opposite nouns), meronymy (part-whole), polysemy (a word that has different meanings), and concept tasks, while sentence level includes sentence formulation, completion tasks, and repair tasks. However, in this way, lexical relations, as well as grammatical semantics, are only assessed without considering the assessment of the child’s lexeme. Lexeme figures out the size and type of the vocabulary or the “language vocabulary knowledge” [12].

There were earlier trials and tests worldwide in different languages that assess semantic development in children. However, the most famous and commonly used and translated are the Expressive One-Word Picture Vocabulary Test “EOWPVT” [14], the Peabody Picture Vocabulary Test “PPVT” [15], and the Receptive One-Word Picture Vocabulary Test “ROWPVT” [16]. For Arabic-speaking societies, the current Arabic language tests were developed to assess the semantics as a part of the assessment of the whole language components such as the Modified Preschool Language Scale–4 (The Arabic version) “The Modified PLS-4” [17], Arabic language test [18]. Semantics was not comprehensively assessed throughout these tests; however, these tests were just screening for semantics deficits in children with delayed language, without stating norms for semantic development.

The importance of semantics is that in the early stages of language development, children understand more words than they produce, children begin to show an understanding of words as early as 9 months of age, and, however, they start producing words at about 12 months [13]. The child’s knowledge of word meaning should be evaluated when she/he is observing the world around him and talking about it [18]. Thus, the appropriateness of any given test for use in evaluation depends upon the proper match between the degree to which the test has characteristics of objectivity, sensitivity, reliability, and validity and the extent of the need for these characteristics by the evaluation process [19]. From the above, this study aimed to construct a test that assesses the pattern of semantic development in Arabic-speaking preschool children aged from 2 to 4 years old. Testing semantic development in preschool children with delayed language development in the Egyptian Arabic language inspired by the Egyptian Arabic environment is necessary for constructing a more efficient therapy program tailored for each child according to his/her language deficits and thus getting a better efficient outcome in the shortest time.

## Methods

The current study was a cross-sectional descriptive study that aimed to construct a test assessing semantics in the Arabic language, the Arabic semantic test (AST). The AST passed through the following stages: (1) test design, (2) test application, and (3) test reliability stage.

### Test design

The AST was prepared in the Colloquial Egyptian Arabic dialect by 3 expert phoniatricains with a least 10 years of experience in the field of phoniatrics. The test was designed guided by the classification and development of semantics according to Cruce [3], as well as the experience of the authors. The test pictures were obtained by searching images on free websites using the Google browser. All the pictures were selected from the Egyptian Arabic culture to be familiar for the children to recognize it. It was presented in familiar bright-colored pictures, and the target questions were articulated in the Colloquial Egyptian Arabic language. Before applying the test to the selected study sample, the test was applied to 20 randomly selected typically developing children in the same age range as the test sample (2–4 years) to ensure that there was no difficulty in understanding the test questions or in recognizing the test pictures. Most children found difficulty in recognizing one picture, and this target picture was replaced by another one.

**Fig. 1:**
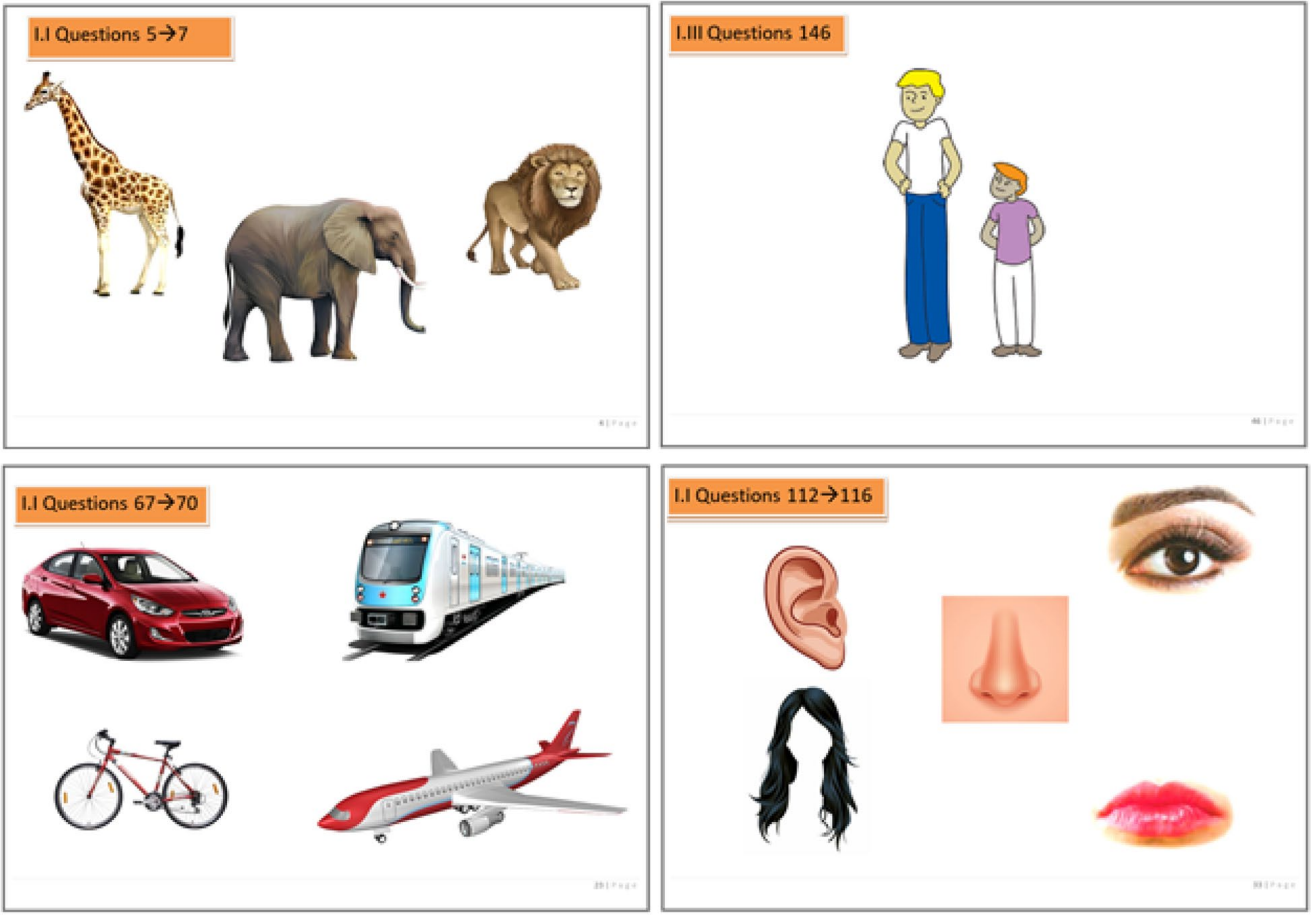
Examples of the test pictures presented to the child. These are 4 pages; each page is presented to the child separately as colored pictures in A4 paper size and landscape orientation

The AST consists of 5 subtests: (1) Identification and/or labeling of words under the major lexical semantic classes namely; nouns, verbs, and adjectives; (2) identification and/or labeling of basic concepts (time, quantity, change, and space concepts); (3) identification and/or labeling of the name of the prototype of 3 semantic classes (toys, food, animals); (4) categorization of 3–5 words under 3 given semantic classes (kitchen utensils, clothes, fruits); and (5) identification of word relations (Polysemy, meronymy, antonymy, and reverse). These 5 subsets were presented in two aspects: the receptive and expressive aspects. Each question in the receptive subdivision has its corresponding questions in the expressive subdivision. Each examined item was presented on one page having minimally 2 pictures and maximally 5 pictures that were shown to the child at one time and used for assessing both receptive and expressive aspects of that item (Fig. 1). The child is supposed to point to or label items she/he asked for according to each question.

### Participants

This study included a hundred and twenty typically developing Egyptian Arabic-speaking children with an age range between 2 and less than 4 years old. The participating children represented different social classes and were randomly selected from different nurseries and preschools in Cairo, Egypt. The children were classified into 4 age groups, with a 6-month age interval between each group. Each group included 30 (15 females, 15 males) children. Children selected to have an average IQ and normal mental age as assessed by the screening test of the Stanford-Binet Intelligence Scale, Arabic version (5th edition) [20]. Children were checked to have normal peripheral hearing. Age-appropriate children with language and/or speech delays were subsequently excluded from the study. Children whose parents used a foreign language (other than the Arabic language) to communicate with their children were excluded to ensure that the test was conducted over native Egyptian Arabic-speaking children and to avoid any bias in the results.

### Test application

The test was applied for all participating children by the same examiner who was a native Egyptian Arabic-speaking phoniatrician, and the test questions are all asked in Colloquial Egyptian Arabic dialect. Before the application of the test, the examiner gave instructions to the child and allows him/her to practice the instructions before starting the actual testing. During the assessment of younger children (below the age of 3 years), the examiner may need to allow parents/caregivers’ participation with the instruction not to reword directions to the child or give the child cues to get the right answer.

Pictures used for assessing the receptive and expressive aspects of the AST were the same and the two aspects were assessed simultaneously. The examiner did not give the child any clues or prompts to answer the questions (either identify or label pictures). This is to ensure that the child acquired the word’s name in his/her lexicon as s/he could easily identify/label it. So, firstly, the child was asked to label pictures of the expressive aspect shown to him/her by the examiner. If the child was not able to label these pictures, the examiner shifted to the receptive aspect and asked the child to point to the picture of the word the examiner told him/her. Applying the test this way will shorten the test time, and keep the child concentrated as much as possible.

### Test timing and scoring

Administration time varies, depending on the child’s age and his or her cooperation during the test. The average test time is 45 min to 1 h. Test scoring was done in the same test set, and it took less than 15 min to calculate the final scores. The child was given 1 point for each correct answer and 0 point for the incorrect answer. The scores were divided into the score of the receptive parts and expressive parts and the total score (summation of scores of both receptive and expressive parts). The total score of the AST is 378 (189 for receptive items and 189 for expressive items). The results of the test scores are presented in tables of 25th, 50th, 75th, and 90th percentiles to determine the level of semantic development of the child in comparison with children of his/her same age.

### Test reliability

The reliability of the total score and the score of each subtest of the AST was estimated by retesting 36 children randomly selected from the already participated children. Each child was assessed twice with a 2-week interval between the 2 assessments.

### Statistical measures and analysis

Data were collected, revised, coded, and analyzed by the Statistical Package for Social Science (IBM SPSS) version 23. Means and standard deviation values were used to describe the quantitative data, and numbers and percentages were used to describe the qualitative data. The analysis of one variance “ANOVA” test and post hoc test were used to compare the results of the 4 age groups, with a *p*-value significant at < 0.05 and a *p*-value significant at < 0.05 and highly significant at < 0.01. Pearson correlation coefficient was used to correlate the children’s chronological ages to the scores of the receptive and expressive aspects of the lexical-semantic subtest of the AST, with a *p*-value that is significant at < 0.05 and highly significant at < 0.01. Mann-Whitney test was used to compare two quantitative parameters with the nonparametric distribution. The comparison between more than two independent groups with quantitative data and nonparametric distribution was done by using the Kruskal-Wallis test followed by post hoc analysis using the Mann-Whitney test when significant.

Cronbach’s alpha test was used to analyze the test-retest reliability of the test-retest and the internal consistency between all the AST subtests. High reliability was considered if Cronbach *α* > 0.75. Test validity was assessed by constructed validity using factor analysis of two tests (KMO measure of sampling and Bartlett’s test of sphericity) which were used for factor analysis of the AST data and was done by principal component analysis. The component matrix explains the variables that lead to the validity of the AST and groups them into components that reflect both common and unique variances of the test variables and how they are related to each other. Two components have been extracted from this model which evaluates the validity of the AST. Component 1 accounts for 61.5%, while component 2 accounts for 73.4%. This means that the variables are measured without errors, so the AST is considered valid.

## Results

An interpretation of the scoring results of the AST is presented in 3 sections: (1) “Results of test application,” (2) “Test reliability,” and (3) “Test validity.”

### Results of test application

Table 1 shows the demographic data of the participating children. Normative data for the AST, means and standard deviations for the total score, receptive and expressive scores, and all the test subtests among the 4 age groups are shown in Table 2. According to one-way ANOVA (*p* < 0.01 is highly significant, *p* < 0.05 is significant), children in group 1 (aged 2 to < 2.6 years) when compared to children in groups 2, 3, and 4 showed statistically significantly lower mean scores regarding the scores of the receptive, expressive parts, and the total test scores (*p*-value < 0.01). Also, children in group 2 (aged 2.6 to < 3 years) when compared to children in groups 3 and 4 showed statistically significantly lower mean scores about the scores of receptive and expressive parts and the total test scores (*p*-value < 0.01) (Table 2).

**Table 1:** Demographic data of the participated children among the 4 age groups

Test groups		Group 1 (2–< 2.6 y) (*n* = 30)	Group 2 (2.6–< 3 y) (*n* = 30)	Group 3 (3–< 3.6 y) (*n* = 30)	Group 4 (3.6–< 4 y) (*n* = 30)
**Age, in years (mean ± SD)**		2.3 (±0.2)	2.9 (±0.1)	3.3 (±0.2)	3.8 (±0.1)
**Gender (no. %)**	**Males**	13 (43.3%)	16 (53.3%)	11 (36.7%)	14 (46.7%)
	**Females**	17 (56.7%)	14 (46.7%)	19 (63.3%)	16 (53.3%)

**Table 2:** Mean and standard deviation (SD) values of all scores of the Arabic semantic test (scores of the total, receptive, and expressive parts) among the 4 age groups and comparison between them using the one-way ANOVA test

Test parts	Total scores	Test scores among the studied 4 age groups (mean ± SD)				***t***-score	***p***-value
		Group 1 (2–< 2.6 y) (*n* = 30)	Group 2 (2.6–< 3 y) (*n* = 30)	Group 3 (3–< 3.6 y) (*n* = 30)	Group 4 (3.6–< 4 y) (*n* = 30)		
**Receptive semantics**	**189**	142.10 ± 30.70	171.27 ± 10.14	176.90 ± 7.77	182.10 ± 4.15	49.38	0.000**
**Expressive semantics**	**189**	90.33 ± 35.66	127.37 ± 17.41	144.30 ± 19.50	153.00 ± 14.55	58.12	0.000**
**Total test score**	**378**	232.43 ± 63.56	297.35 ± 27.02	317.99 ± 27.06	332.61 ± 15.37	9.30	0.000**

ANOVA test: **p-value is highly significant at < 0.01

**Fig. 2:**
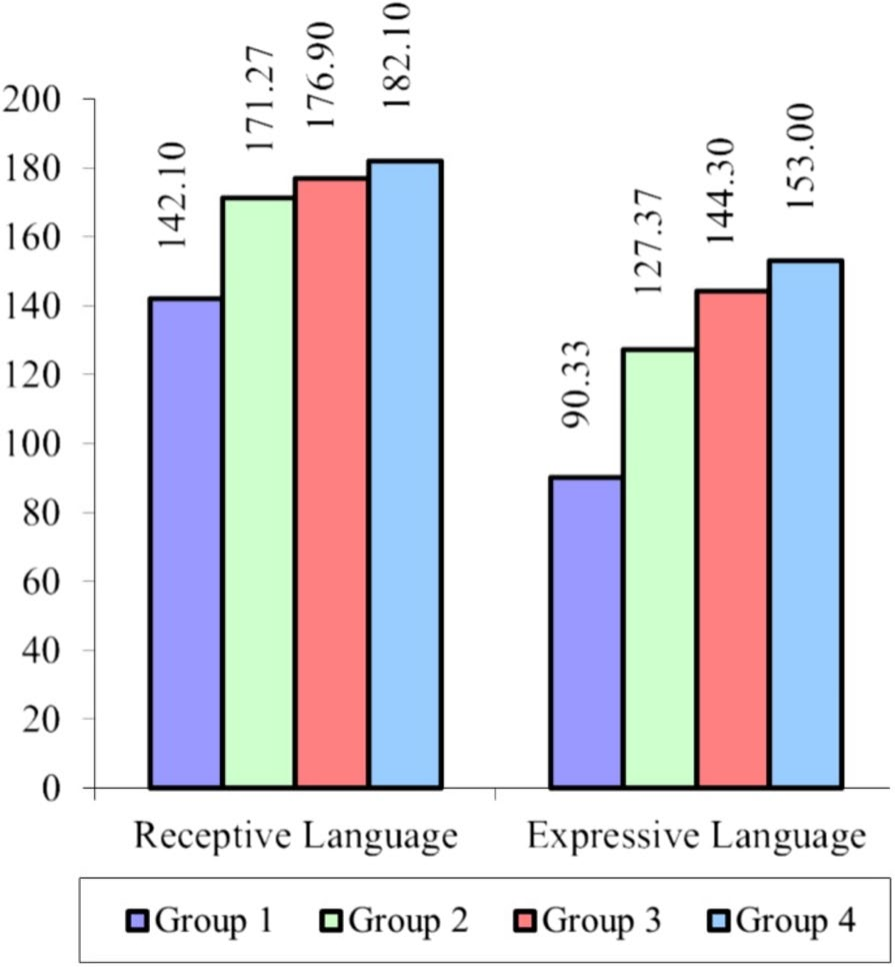
Comparison between the 4 participating age groups as regards the mean total score of the receptive and expressive parts of the Arabic semantic test among the 4 participating age groups, using the one-way analysis of one variance “ANOVA” test

According to the post hoc test (Table 3 and Fig. 2), there is a statistically significant difference between group 1 when compared to either groups 2, 3, or 4 as regards (total, receptive, and expressive semantic scores). Also, there is no statistically significant difference when comparing group 2 to group 3 as regards the receptive score. However, there is a high statistical significance as regards expressive scores with a *p*-value equal to 0.006 (*p*-value < 0.01 is highly significant). Besides, there is a statistically significant difference between group 2 and group 3 as regards total test scores with a *p*-value equal to 0.037 (significant *p*-value is at < 0.05). There is a statistically significant difference between group 2 and group 4 regarding the scores of receptive scores and a highly statistically significant difference as regards expressive, and the total scores of the test, with a *p*-value of 0.000 (*p*-value < 0.01 is highly significant). There is no statistically significant difference between group 3 and group 4 regarding the scores of the receptive, expressive, and total scores of the test.

**Table 3:** Post hoc analysis and multi-comparison between the 4 participating age groups as regards scores (total, receptive, and expressive) of the Arabic semantic test

Post hoc analysis and multi-comparison between the 4 age groups						
Parameters	Group 1 vs 2	Group 1 vs 3	Group 1 vs 4	Group 2 vs 3	Group 2 vs 4	Group 3 vs 4
Receptive semantics	0.000**	0.000**	0.000**	0.195	0.014*	0.232
Expressive semantics	0.000**	0.000**	0.000**	0.006**	0.000**	0.150
Total test score	0.000**	0.000**	0.000**	0.037*	0.000**	0.138

Post hoc test: *p-value is significant at < 0.05, **p-value is highly significant at < 0.01

Table 3 and Fig. 2 show that children in group 3 when compared to children in group 4 showed statistically significantly lower mean scores as regards the reception of the time concept and quantity concept (items of the receptive part). There was also a statistically insignificant difference in expressing the time concept (an item of the expressive part) among the 4 studied groups (its mean score is nearly the same among the 4 participating age groups) (Table 4). This shows that the expressing time concept is not developed before the age of 4 years (the maximum age of the participated children) (Table 5).

**Table 4:** Mean scores and standard deviation scores of each item of the receptive part of the Arabic semantic test among the 4 participating age groups and comparison between them using the one-way ANOVA test

Items of the receptive semantic	Total scores	Test scores among the studied 4 age groups (mean ± SD)				***t***-score	***p***-value
		Group 1 (2–< 2.6 y) (*n* = 30)	Group 2 (2.6–< 3 y) (*n* = 30)	Group 3 (3–< 3.6 y) (*n* = 30)	Group 4 (3.6–< 4 y) (*n* = 30)		
**I. Identification of lexical semantic classes**	153	125.60 ± 20.49	145.60 ± 5.10	148.03 ± 4.39	150.33 ± 2.48	41.83	0.000**
1) Nouns under different semantic classes	131	108.17 ± 16.34	125.00 ± 4.33	126.50 ± 4.26	128.50 ± 2.27	21.78	0.000**
2) Action verbs	12	10.60 ± 1.83	11.73 ± 0.58	11.83 ± 0.46	11.97 ± 0.18	37.88	0.000**
3) Adjectives	10	6.83 ± 3.27	8.87 ± 1.43	9.70 ± 0.70	9.87 ± 0.35	36.35	0.000**
**II. Identify basic concepts**	18	6.50 ± 5.82	10.90 ± 4.66	13.03 ± 3.27	15.27 ± 2.00	42.74	0.000**
1) Time concept	4	1.60 ± 1.83	2.53 ± 1.31	2.63 ± 1.22	3.33 ± 0.88	16.33	0.001**
2) Quantity concept	3	0.80 ± 1.16	1.40 ± 1.28	1.87 ± 1.11	2.60 ± 0.72	31.84	0.000**
3) Change concept	7	2.60 ± 2.74	3.97 ± 2.47	5.10 ± 1.63	5.50 ± 1.43	19.82	0.000**
4) Space concept	4	1.50 ± 1.14	3.00 ± 1.41	3.43 ± 1.04	3.83 ± 0.46	53.53	0.000**
**III. Identify the name of a certain prototype**	3	2.13 ± 0.97	2.90 ± 0.31	3.00 ± 0.00	2.90 ± 0.31	38.75	0.000**
**IV. Categorization**	3	1.47 ± 1.46	2.53 ± 0.68	2.77 ± 0.63	2.93 ± 0.25	28.37	0.000**
**V. Word relations**	12	6.40 ± 4.61	9.33 ± 1.94	10.07 ± 1.98	10.67 ± 1.49	18.54	0.000**
1) Polysemy	8	4.63 ± 3.01	7.07 ± 1.14	7.30 ± 1.09	7.53 ± 0.97	27.23	0.000**
2) Meronyms	4	1.77 ± 1.81	2.27 ± 1.20	2.77 ± 1.22	3.13 ± 1.22	13.23	0.004**
**Test receptive scores**	189	142.10 ± 30.70	171.3 ± 10.2	176.90 ± 7.77	182.10 ± 4.15	49.38	0.000**

ANOVA test: *p-value is significant at < 0.05, **p-value is highly significant at < 0.01

**Table 5:** Mean scores and standard deviation scores of each item of the expressive part of the Arabic semantic test among the 4 participating age groups and comparison between them using the one-way ANOVA test

Items of the expressive semantic	Total scores	Test scores among the studied 4 age groups (mean ± SD)				***t***-score	***p***-value
		Group 1 (2–< 2.6 y) (*n* = 30)	Group 2 (2.6–< 3 y) (*n* = 30)	Group 3 (3–< 3.6 y) (*n* = 30)	Group 4 (3.6–< 4 y) (*n* = 30)		
**I. Identification of lexical semantic classes**	**153**	83.1 ± 30.4	112.9 ± 12.9	125.5 ± 15.2	132.6 ± 11.5	55.444	0.000**
1) Nouns under different semantic classes	**131**	73.2 ± 25.2	98.8 ± 11.6	107.7 ± 13.2	114.33 ± 9.7	32.660	0.000**
2) Action verbs	**12**	7.03 ± 3.5	9.4 ± 1.6	10.8 ± 1.4	11.00 ± 1.02	54.671	0.000**
3) Adjectives	**10**	2.9 ± 3.17	4.8 ± 2.2	7.0 ± 1.9	7.30 ± 2.12	42.655	0.000**
**II. Identify basic concepts**	**8**	1.2 ± 1.56	2.4 ± 1.5	3.3 ± 1.9	3.27 ± 1.62	23.594	0.000**
1) Time concept	**4**	0.6 ± 1.28	0.7 ± 0.8	1.3 ± 1.4	1.00 ± 1.34	5.772	0.123
2) Space concept	**4**	0.6 ± 0.86	1.7 ± 1.1	2.0 ± 1.11	2.27 ± 0.69	37.352	0.000**
**III. Identify the name of a certain prototype**	**3**	0.3 ± 0.80	0.6 ± 0.7	1.6 ± 1.2	1.73 ± 1.11	35.328	0.000**
**IV. Categorization**	**3**	0.00 ± 0.00	0.3 ± 0.7	1.03 ± 1.1	1.27 ± 1.11	36.410	0.000**
**V. Word relations**	**22**	5.70 ± 5.94	11.1 ± 4.9	12.9 ± 3.7	14.10 ± 3.27	35.102	0.000**
1) Antonyms	**11**	2.60 ± 3.04	4.9 ± 2.8	5.3 ± 1.8	6.33 ± 1.97	33.584	0.000**
2) Reverse verbs	**11**	3.10 ± 3.08	6.2 ± 2.72	7.6 ± 2.4	7.77 ± 1.77	36.617	0.000**
**Test expressive scores**	**189**	90.33 ± 35.66	127.4 ± 17.4	144.3 ± 19.5	153.0 ± 14.5	58.119	0.000**

ANOVA test: *p-value is significant at < 0.05, **p-value is highly significant at < 0.01

The ranks of the 25th, 50th, 75th, and 90th are shown in Tables 6 and 7. Semantic development is considered poor with total, receptive, and expressive scores less than the 25th percentile, fair with scores between the 25th and 50th percentiles, good with scores between the 50th and 75th percentiles, and excellent with scores equal to or higher than the 90th percentiles.

**Table 6:** The ranks of the 25th, 50th, 75th, and 90th percentiles of the total scores of the Arabic semantic test among the 4 participating age groups

AST scores		Group 1 (2–< 2.6 y) (*n* = 30)			Group 2 (2.6–< 3 y) (*n* = 30)			Group 3 (3–< 3.6 y) (*n* = 30)			Group 4 (3.6–< 4 y) (*n* = 30)		
		Receptive	Expressive	Total	Receptive	Expressive	Total	Receptive	Expressive	Total	Receptive	Expressive	Total
**Percentile ranks**	**25th**	119	60	184	163	116	280	174	131	308	180	141	321
	**50th**	149	86	230	173	124	297	178	142	321	182	154	337
	**75th**	167	119	298	179	144	323	182	157	339	185	164	346
	**90th**	182	143	312	183	154	337	186	163	354	188	173	357

**Table 7:** The ranks of the 25th, 50th, 75th, and 90th percentiles of the scores of each item of the receptive and expressive part of the Arabic semantic test among the 4 participating age groups

Items of the receptive and expressive parts		Total scores	Ranks of percentiles of the receptive and expressive parts among the 4 studied age groups															
			Group 1 (2–< 2.6 y) (*n* = 30)				Group 2 (2.6–< 3 y) (*n* = 30)				Group 3 (3–< 3.6 y) (*n* = 30)				Group 4 (3.6–< 4 y) (*n* = 30)			
			25	50	75	90	25	50	75	90	25	50	75	90	25	50	75	90
**I. Identification of lexical semantic classes**	**Receptive**	**153**	112	130	144	151	143	147	150	152	147	150	151	152	150	151	152	152
	**Expressive**	**153**	60	79	110	127	107	111	120	133	116	127	136	146	124	135	142	146
**II. Identify basic concepts**	**Receptive**	**18**	0	7	11	14	6	13	14	15	12	13	15	17	14	15	17	18
	**Expressive**	**8**	0	0	2	4	2	3	3	5	2	3	4	6	2	3	4	6
**III. Identify the name of a certain prototype**	**Receptive**	**3**	2	2	3	3	3	3	3	3	3	3	3	3	3	3	3	3
	**Expressive**	**3**	0	0	0	1	0	1	1	2	1	2	3	3	1	2	3	3
**IV. Categorization**	**Receptive**	**3**	0	2	3	3	2	3	3	3	3	3	3	3	3	3	3	3
	**Expressive**	**3**	0	0	0	0	0	0	0	2	0	1	2	3	0	1	2	3
**V. Word relations**	**Receptive**	**12**	3	8	11	12	8	10	11	12	9	10	12	12	10	11	12	12
	**Expressive**	**22**	0	5	10	10	8	13	14	20	10	15	15	17	12	14	17	18
**Total scores**	**Receptive**	**189**	119	149	167	182	163	173	179	183	174	178	182	186	180	182	185	188
	**Expressive**	**189**	60	86	119	143	116	124	144	154	131	142	157	173	141	154	164	173

Table 8 shows the results of the Arabic semantic test indicating the development of semantics among children from 2 years to less than 4 years. By application of AST to participating children in this study, it was revealed that *lexical-semantic*, including noun classes, verbs, and adjectives, develops as early as the age of 2 years. Noun classes and verbs started to be identified by children at the age of 2 years. As children grow, noun vocabulary increases both expressively and receptively. Adjectives started to be acquired at age of 2.6 to 3 years and were mostly labeled by the age of 3 to 3.6 years. Regards the *identification of basic concepts* begins to appear at the age of (2.6 – 3 years) as the child can understand some of the time concepts (day and night), the concept of color change, and the place indicators (above and under). By the age of 3–3.6 years, children started to identify receptively the other time concepts (such as day and night), the concept of quantity (more than), the concept of change in size, and other concepts of place (such as beside and in-between). The concept of summer and winter and the more complex preposition are better understood receptively by the age of 3.6 to 4 years. Before the age of 4 years, children could only label place indicators and use them correctly in their speech.

**Table 8:** Results of the Arabic semantic test indicating the development of semantics among children from 2 years to less than 4 years

			2–< 2.6 years	2.6–< 3 years	3–3.6 years	3.6–< 4 years
**I. Identification of lexical semantic classes**	**Nouns under noun classes**	Receptive	All family members except sister and brother, farm animals, food kinds, body parts, vegetables, and fruits, daily used common objects, clothing items (shoes, socks, jeans), places, wild animals (giraffe, lion, and elephant)	Family members, wild animals (giraffe, lion, elephant, and monkey), birds, plants, insects, vegetables and fruits, clothing items, food, electronic devices, furniture, common daily used objects, body parts, colors, places (house, street, sea, playground) Some means of transportation (car, bicycle, airplane). Some jobs (doctor, soccer mainly)	All means of transportation, electronic devices, places, jobs, places Most colors (red, yellow, blue, white, black, jobs (soccer, doctor, policeman)	Differentiate between birds (chicken and roaster), reptiles, plants, and all jobs
		Expressive	Some family members (mother and father) and grandparents if they are alive Some farm animals (sheep, cats, dogs, and horses), some food kinds. Body parts (mouth, eyes, hands, and feet) Some daily common objects (plate, spoon, money, and keys) Wild animals (lion only), vegetables and fruits (cucumber, grapes, and banana only), means of transportation (care only), plants (flower), and fish Label little of verbs (drink, bath, comb)	All farm animals, fruits, clothing items, food kinds, common objects, and body parts, farm animals, fruits, food, clothing items, electronic devices (mobile, fan, washing machine), furniture (chair, table, door, bed) Some wild animals (giraffe, lion, elephant), some vegetables, some colors (red, blue, yellow, and black mainly), and some means of transportation (car, airplane, bicycle) Birds (duck mainly), insects (butterfly and fish), plants, places (sea only)	All family members (sister and brother) if they have any, wild animals and farm animals, food kinds, vegetables, means of transportation, furniture, electronic devices, colors, plants (flower and tree) Some birds (mainly ducks, birds, and chickens) Little insects (butterfly and fish), little places (sea, a house only), jobs (doctor only)	Most insects and reptiles Some of the places (house, street, and sea)
	**Verbs**	Receptive	Most action verbs	All action verbs	All action verbs	All action verbs
		Expressive		Labels some of it (comb, play, eat, drink, wash, play)	Labels most of them	Labels all action verbs
	**Adjectives**	Receptive	None	Most adjectives (big, dirty, beautiful, cold, clean, ugly, hot)	Knows all adjectives	Knows all the adjectives
		Expressive	None	None	Labels some of them (big, small, beautiful, hot)	Labels most of them (big, small, beautiful, clean, ugly, hot)
**II. Identify the name of a certain prototype**		Receptive	Identify 2 prototypes (food and animal)	Identify 2 prototypes (food and animal)	Identify all prototypes	Identify all prototypes
		Expressive	None	None	Identify prototype (toys)	Identify the prototype (toys and food)
**III. Identification of basic concepts**		Receptive	None	Concept of color change, time concept (day and night), some of the place indicators (above and under)	Concept of quantity (more than), size change, and all place indicators	Time concept (summer and winter), the more complex preposition (beside and in-between) is better understood
		Expressive	None	Can label the place indicators (above and under)	None	Can label place indicators (above and under, beside and in-between)
**IV. Categorization**		Receptive	None	Can group clothes and fruits	Can group all objects and identify their prototype	Can group all objects and identify their prototype
		Expressive	None	None	Could group (clothes)	Could group (clothes and fruits)
**V. Word relations**		Receptive	None	Understands some polysemy	Understand polysemy, some meronyms	Understand polysemy and some meronym
		Expressive	None	Some antonyms (up and down, small, and big), some reverse verbs (laugh and cry, wakes up and sleep)	Most reverse verbs	Most antonyms (up and down, small, big, sick, and healthy) and most reverse verbs

As early as the age of 2 to 2.6 years, children participating in this study could understand and identify the name of the semantic groups (prototype name), namely food and animals, but cannot label them. However, they were able to sort (categorize) things under the same semantic group by the age of 2.6 to 3 years. They could group objects under clothes and fruit groups only. As they grew to 3–3.6 years, they were able to group all objects and identify their prototype name (name of the semantic group), but still, they cannot do that expressively until the age of 3.6–4 years. By the age of 2.6 to 3 years, children begin to understand the *word relations*, starting by understanding polysemy, labeling some antonyms (up and down, small and big) and some reverse verbs (laugh and cry, wakes up and sleep). Meronyms begin to be understood at the age of 3 to 3.6 years. Other difficult antonyms (like full and empty, sick and healthy) and most word relations could be identified by the age of 3 to 3.6 years and labeled by the age of 3.6 to 4 years.

### Test reliability

The high value of the Cronbach *α*-test for testing test-retest reliability of the total score (0.864; > 0.70), the score of receptive (0.827; > 0.70), and expressive parts (0.832; > 0.70) indicates the high reliability of the AST as an assessment tool of semantics development among Egyptian Arabic-speaking children (Table 9). In addition, the results of the Mann-Whitney test revealed no difference between answers if the test is repeated twice (*p*-value > 0.813; significant *p*-value is < 0.05). This indicates high reliability to use this test in the assessment of semantics (Table 10). The high internal consistency between all the AST subsets is reported by the high value of the Cronbach *α* reliability analysis (0.987; > 0.75) (Table 11).

**Table 9:** The Alpha Cronbach for the test-retest reliability analysis of the items of the Arabic semantic test

The Arabic semantic test			
**Total number of cases**	**120**		
**Number of variables**	**Receptive part**	**Expressive part**	**Total test**
	189	189	378
**Alpha Cronbach**	0.935*	0.927*	0.984*
**Cronbach’s alpha based on standardized items**	0.945	0.939	0.987

*Alpha Cronbach (> 0.75) indicates high reliability and high internal consistency of AST

**Table 10:** Correlation between the scores test-retest reliability of the Arabic semantic test by Mann-Whitney test

Test scores (mean ± SD)	First measurement (***n*** = 36)	Second measurement (***n*** = 36)	Test value	***p***-value
**Receptive part**	168.89 ± 21.93	169.28 ± 22.23	−0.287	0.774*
**Expressive part**	129.36 ± 35.15	132.14 ± 37.01	−0.524	0.600*
**Total test score**	298.25 ± 55.60	301.42 ± 57.73	0.237	0.813*

Mann Whitney test: *p-value is at (> 0.05) indicates the high reliability to use the AST in assessment of semantics

**Table 11:** The Alpha Cronbach reliability analysis for the reliability of the used tool “the Arabic semantic test”

The Arabic semantic test			
**Total number of cases**	**36**		
**Number of variables**	**Receptive part**	**Expressive part**	**Total test**
	189	189	378
**Alpha Cronbach**	0.827*	0.832*	0.856*
**Cronbach’s alpha based on standardized items**	0.874	0.883	0.864

Alpha Cronbach > 0.75. This indicates high reliability to use this test in the assessment of semantics

### Test validity

The Kaiser-Meyer-Olkin (KMO) measure of sampling adequacy is a statistic that indicates the proportion of variance in test variables that might be caused by underlying factors. High values (close to 1.0) generally indicate that factor analysis may be useful with this study data. If the value is less than 0.50, the results of the factor analysis probably will not be extremely useful. Table 12 shows that the sample sufficiency index ΚΜΟ by Kaiser--Meyer-Olkin, which compares the sizes of the observed correlation coefficients to the sizes of the partial correlation coefficients for the sum of analysis variables is 88.6%, and it is reliable because it overcomes 70% by far. Besides, the supposition test of sphericity by the Bartlett test is rejected on a level of statistical significance *p* < 0.0005 for approx. chi-square = 870.648. Consequently, the coefficients are not all zero, so the second acceptance of factor analysis is satisfied. As a result, “Bartlett’s test of sphericity” tests the hypothesis that the correlation matrix is an identity matrix, which would indicate those study variables are unrelated and therefore unsuitable for structure detection. Small values (less than 0.05) of the significance level show that factor analysis may be useful with study data. Acceptances for the conduct of factor analysis are satisfied. This indicates the high validity of the AST.

**Table 12:** Test validity using factor analysis by KMOAQ4 and Bartlett’s test of sphericity

Kaiser-Meyer-Olkin measure of sampling adequacy (KMO)		0.886
**Bartlett’s test of sphericity**	**Approx. chi-square**	870.648
	**Df**	45
	**Sig.**	0.000*

^*^p-value is significant at < 0.05

## Discussion

Semantics is an important brick in language construction. Understanding the basic intrinsic rules of semantics comes along with language acquisition. This allows the speakers to string words together in the correct linguistic order in the sentences, so listeners could easily derive meaning from those sentences. Semantics is the language content that conveys the meaning and helps the child understand and label the world around him. Lower gain of language vocabulary is the first sign of delayed language development among preschool children. Over the last years with the increased time of children’s exposure to screens (mobiles, tablets, and electronic games devices), children’s vocabulary development, hence language development is affected especially with the lockdown during the COVID-19 pandemic [21]. Among studies that reported the effect of screens on children’s language children is a study by Bergmann et al. [22]. Authors reported that children who have had more screen time during lockdown were reported to show smaller gains in vocabulary development during the lockdown, separately for children’s receptive and expressive percentile scores. These raised the need for a test that could address early identification of any vocabulary delay, and upon its results, a language rehabilitation program could be provided according to the child’s needs.

This study presents the first comprehensive Arabic test that assesses semantics with its subcategories: the AST. AST is an objective test that can be used as a diagnostic and prognostic tool. AST gives a precise estimate of the child’s receptive and expressive vocabulary, as well as the child’s understanding of concepts.

AST, as a standardized assessment tool, helps in constructing an objective therapy plan adequate to children’s needs and deficits and is used to follow up on children’s progress throughout their rehabilitation program. Moreover, AST is an important prognostic tool for children with language delays. The AST scoring system allows a comparison of a child’s semantic development to his/her mates of the same age. This helps phoniatricains and speech-language pathologists to decide the size of gap deficits of the language-delayed child and in turn determine the child’s prognosis across the therapy plan.

The currently used Arabic language tests measure semantic comprehension as a part of a comprehensive test screening or assessing the development of all language domains (semantics, syntax, phonology, pragmatics) such as the language test by Kotby et al. [18], the modified preschool language screening test — the Arabic version [17], the Receptive-Expressive Arabic Language Scales (REAL scale), and the “A Proficient Preschooler Language Evaluation Tool” (APPLE tool) by Osman [23, 24]. Unlike these tests, AST is a holistic test that provides reliable information about the semantics of Egyptian Arabic-speaking children. It is not a screening test like the Peabody Picture Vocabulary test “PPVT-4” or modified PLS-4, but it is an objective tool that depends on interviewing and observing the child him/herself. AST assesses semantic development on the receptive and expressive levels. Therefore, it can be used to assess the amount of vocabulary knowledge recognized (receptive or passive vocabulary) by younger children with still little active vocabulary.

Although AST appears to be similar in structure to the PPVT-4, the two tests are different. The PPVT-4 test is a screening test that assesses only the receptive semantics; however, AST assesses semantics both receptively and expressively. Besides, AST addresses a further parameter compared to the PPVT-4, namely the conceptual semantics and word relations on both receptive and expressive levels. Among the recent tests used to assess semantics among English-speaking children are the Expressive One-Word Picture Vocabulary Test–4 (EOWPVT-4) and the Receptive One-Word Picture Vocabulary Test-4 (ROWPVT-4) [15, 16]. Unlike AST, the Receptive One-Word Picture Vocabulary Test gives information on a child’s lexical knowledge on the receptive level only, and the Expressive One-Word Picture Vocabulary Test counts the amount of expressive vocabulary.

Also, the abovementioned tests are heavily loaded with culturally, socially, and linguistically restricted contents, which could not be translated for assessing Arabic-speaking children. For that reason, there was a need to create a comparable test to study semantic development where the test items were grabbed from the Egyptian Arabic language with culturally, socially, and linguistically suitable items. The AST questions and pictures were constructed to suit the Arabic-speaking children and Arab culture. It uses common environmental settings familiar to any Arabic child.

A crucial and yet complex component of a child’s language development is the acquisition of the lexicon. Moreover, lexical-semantic development also interacts with acquisition processes in other linguistic domains [25]. In this study, preschool children (between 2 years to less than 4 years) were selected to study their semantic development. This is because semantic development is particularly rapid in preschool years as reported by Mengisidou et al. [26]. Moreover, there is little to know about the developmental trends in semantic fluency at preschool age of Arabic-speaking children. It would be helpful to know the developmental trajectory of semantic fluency in preschool-typically developed children. That is why this small age range was chosen while constructing the AST.

As a holistic test, the AST was visualized as a collective semantic information tool and designed to assess the semantic development across 4 major semantic domains: identification and labeling of lexical semantics, identification of basic concepts, identification of the name of a certain prototype and categorization, and identification and using different word relations (polysemy, meronym, antonyms, and reverse verbs). These 4 domains were chosen to complete the whole semantic picture depending on Cruce’s division of semantics. Cruce [3] divided semantics into three subfields: lexical semantics, grammatical semantics, and conceptual semantics (logical semantics). Lexical and conceptual semantics was the main field of interest in the construction of the AST, because vocabulary is a useful proxy for general verbal language skills. Certainly, data from typical development indicates that vocabulary correlates highly with other language abilities and is the single best predictor of academic success for children starting school [27]. Lexical semantics focuses on “content” words rather than “grammatical” words, and conceptual semantics refers to word meaning in conceptual structure [28]. The central principle of Jackendoff’s conceptual semantics is that describing meaning involves describing mental representations. For him, the semantic structure is a conceptual structure. Jackendoff held the idea that humans’ conceptual structure is built up of units such as conceptualized physical objects, events, properties, times, quantities, and intentions. These conceptualized objects are in our minds and determine our perception of the world.

While grammatical semantics studies aspects of meaning which have direct relevance to syntax, grammatical semantics is assessed comprehensively in other Arabic tests like the modified PLS-4 [17], the REAL scale (morpho-syntax subtest) [23], and the APPLE tool [24]. For that reason, the part about grammatical semantics was omitted from AST; moreover, it is overlapping with syntax.

AST is easy, and child-friendly, with familiar pictures, grabbed from the Egyptian Arabic environment. AST administration is simple, and it does not require the child to read or write because the manner of the individual’s response to stimulus vocabulary is to point in any fashion to one of four pictures that best fits the stimulus work. Both receptive and expressive scales are assessed at the same time where we start with the specific receptive item, and if the child gets, it applies the corresponding expressive item. If the child failed on the receptive item, the corresponding expressive item will not be applied, which apparently will save time and effort.

However, there is no starting or endpoint which makes the test time-consuming as the whole test should be applied for all ages. Making a start and endpoint for semantic knowledge is difficult because the main semantic domains were assessed in the four groups; the difference between the 4 age groups emerged from the weight of semantic knowledge rather than the unacquaintance of specific semantic domains. The four groups scored in all AST subtests, but the score varied according to age and so semantic weight of knowledge.

The time of administration of AST is 40 min to 1 h, which is considered lengthy in comparison with other rapid tests like PPVT-4. However, length of administration can be considered as an advantage from another point of view because children start to be friendly with strangers after spending more time with them; for further investigation of the child’s semantic fluency, a clinician should spend more time with the child to have a greater opportunity of talking to the child.

Based on the notion of the importance of early detection and early intervention of semantic deficits, this study presents a test that could be attributed to routine checks of early semantic abilities among preschool children in the primary care unit. AST could be applied to preschool children (from the age of 2 years and to less than 4 years), the normal values are in the form of percentile ranges and not strict numbers, and this gives a chance to consider the variable capabilities of normal children.

As did mention before, some factors could affect semantic development in children, namely environmental factors and factors related to the child [10]. While in application of AST, it was noticed that children’s performance was significantly affected by parents’ educational level. The scores of children whose parents had a high level of education were better than those having lower-educated parents. This could be explained by the parents with a lower educational level using a more restrictive language instead of an explanatory language, and they usually neglect to build up a language-stimulating environment for their children. Following the restricted language, parents tend to be more directive; they forbid and command more and talk to their children less frequently. This could be less stimulating for language development than the illustrative method for dealing with children which is usually used by highly educated parents who tend to name and verbalize objects and talk more frequently to their children. Also, highly educated parents have higher expectations of their children’s language abilities. On the contrary, some parents of high education were more concerned with teaching their children a second language (mostly English), and it was so remarkable to find some Egyptian children of Egyptian parents to be semantically fluent in English language rather than their Arabic native language. Certainly, children with extreme scores in AST were excluded from the results of this study to avoid any bias. The child’s chronological age affects the results of AST with a statistically significant positive relationship found between the chronological age of the participated children and AST scores, while there was no significant effect of gender on the AST scores. This indicates that male and female children develop semantics with the same profile and the same rate of development, and this matches results by Franklin and Grossman [29], Abo Hassiba [17], and Kotby et al. [18].

From a clinical phoniatric point of view, the clinical importance of AST is an important tool to be used for the assessment of children with language impairment before starting language therapy. Comparing semantics in children with language disorders and typical language-developed children with AST could increase the clinical importance of AST as a tool to diagnose children with language impairment and predict their improvement. Early assessment of semantic abilities by the AST will help a lot in making a decision regarding differential diagnosis of delayed language development and drawing the content and design of early intervention programs that suit every child’s needs and deficits.

## Conclusion

AST is a valid and reliable assessment tool for early semantic development among preschool Arabic-speaking children. AST is directly applied to the children using familiar bright-colored pictures. AST assesses semantics on the receptive and expressive levels and at the word and sentence levels. Further studies are needed to address the efficacy of the AST in planning language rehabilitation programs based on its results.

## Data Availability

The datasets used and/or analyzed during the current study are available from the corresponding author upon reasonable request.

## Abbreviations

APPLE tool: A Proficient Preschooler Language Evaluation Tool
AS: Arabic semantic test
CELF: Clinical Evaluation of Language Fundamentals
EOWPV: Expressive One-Word Picture Vocabulary
PLS: Preschool Language Scale
PPVT: Peabody Picture Vocabulary Test
REAL Scale: Receptive-Expressive Arabic Language Scale
ROWPVT: Receptive One-Word Picture Vocabulary Test
